# Host methylation predicts SARS-CoV-2 infection and clinical outcome

**DOI:** 10.1038/s43856-021-00042-y

**Published:** 2021-10-26

**Authors:** Iain R. Konigsberg, Bret Barnes, Monica Campbell, Elizabeth Davidson, Yingfei Zhen, Olivia Pallisard, Meher Preethi Boorgula, Corey Cox, Debmalya Nandy, Souvik Seal, Kristy Crooks, Evan Sticca, Genelle F. Harrison, Andrew Hopkinson, Alexis Vest, Cosby G. Arnold, Michael G. Kahn, David P. Kao, Brett R. Peterson, Stephen J. Wicks, Debashis Ghosh, Steve Horvath, Wanding Zhou, Rasika A. Mathias, Paul J. Norman, Rishi Porecha, Ivana V. Yang, Christopher R. Gignoux, Andrew A. Monte, Alem Taye, Kathleen C. Barnes

**Affiliations:** 1 School of Medicine, University of Colorado Anschutz Medical Campus, Aurora, CO USA; 2 Illumina, Inc., San Diego, CA USA; 3 Colorado School of Public Health, University of Colorado Anschutz Medical Campus, Aurora, CO USA; 4 University of California Los Angeles, Los Angeles, CA USA; 5 The Children’s Hospital of Philadelphia, Philadelphia, PA USA; 6 Johns Hopkins University, Baltimore, MD USA

**Keywords:** Predictive markers, Viral infection

## Abstract

**Background:**

Since the onset of the SARS-CoV-2 pandemic, most clinical testing has focused on RT-PCR^1^. Host epigenome manipulation post coronavirus infection^2–4^ suggests that DNA methylation signatures may differentiate patients with SARS-CoV-2 infection from uninfected individuals, and help predict COVID-19 disease severity, even at initial presentation.

**Methods:**

We customized Illumina’s Infinium MethylationEPIC array to enhance immune response detection and profiled peripheral blood samples from 164 COVID-19 patients with longitudinal measurements of disease severity and 296 patient controls.

**Results:**

Epigenome-wide association analysis revealed 13,033 genome-wide significant methylation sites for case-vs-control status. Genes and pathways involved in interferon signaling and viral response were significantly enriched among differentially methylated sites. We observe highly significant associations at genes previously reported in genetic association studies (*e.g.* *IRF7*, *OAS1*). Using machine learning techniques, models built using sparse regression yielded highly predictive findings: cross-validated best fit AUC was 93.6% for case-vs-control status, and 79.1%, 80.8%, and 84.4% for hospitalization, ICU admission, and progression to death, respectively.

**Conclusions:**

In summary, the strong COVID-19-specific epigenetic signature in peripheral blood driven by key immune-related pathways related to infection status, disease severity, and clinical deterioration provides insights useful for diagnosis and prognosis of patients with viral infections.

## Plain language summary

Viral infections affect the body in many ways, including via changes to the epigenome, the sum of chemical modifications to an individual’s collection of genes that affect gene activity. Here, we analyzed the epigenome in blood samples from people with and without COVID-19 to determine whether we could find changes consistent with SARS-CoV-2 infection. Using a combination of statistical and machine learning techniques, we identify markers of SARS-CoV-2 infection as well as of severity and progression of COVID-19 disease. These signals of disease progression were present from the initial blood draw when first walking into the hospital. Together, these approaches demonstrate the potential of measuring the epigenome for monitoring SARS-CoV-2 status and severity.

## Abstract (web summary)

Konigsberg et al. profile DNA methylation in blood samples from SARS-CoV-2 cases and controls. The authors use machine learning to classify infected vs. non-infected individuals and predict clinical outcomes related to disease severity.

## Introduction

Coronaviruses (CoV) comprise a large group of human and animal pathogens, including the novel enveloped RNA betacoronavirus referred to as severe acute respiratory syndrome coronavirus 2 (SARS-CoV-2)^5^. This pathogen is associated with coronavirus disease 2019 (COVID-19) first identified in Wuhan, China in 2019^6^ and declared a pandemic on March 11, 2020^7^. Since the onset of the pandemic, multiple tests for diagnosing COVID-19 have been launched, including real-time reverse transcriptase–polymerase chain reaction (RT-PCR), specific antibody detection, and next-generation sequencing assays that query for current or past infections^1^. With the exception of next-generation sequencing, which can discern viral subtypes, most diagnostic tests are viral strain dependent, can carry a high false negative rate, do not discern if the virus is viable and replicating, and do not predict clinical outcomes of infection^1,8,9^. For example, pre-symptomatic patients may test negative^10,11^ while patients who have recovered may continue to test positive though they are no longer infectious^12^. Accurate diagnostics are urgently required to control continued communal spread, to better understand host response, and for the development of vaccines and antivirals^13^.

Individuals infected with SARS-CoV-2 have a variable course of infection, ranging from asymptomatic to death. Although the fatality rate varies tremendously according to demographic characteristics and co-morbidities^14^, the U.S. ranks as one of the countries with the highest COVID-19 mortality rates^15^. Identification of which SARS-CoV-2-infected patients are most likely to develop severe disease would enable clinicians to triage patients via augmented clinical decision support. Having more information on disease severity has recently become critical due to widespread lack of hospital and intensive care unit (ICU) capacity, necessitating difficult decisions about resource triage. To our knowledge, no test can predict COVID-19 clinical course or severity, although work on cytokine abundance ratios after hospitalization has been proposed as a prognostic indicator of severe outcomes^16^.

There is considerable evidence that enveloped RNA viruses such as CoV can manipulate the host’s epigenome via evolved functions that antagonize and regulate the host innate immune antiviral defense processes^2,3^, specifically via DNA methylation. Viral-mediated antagonism of antigen-presentation gene expression in the case of Middle East respiratory syndrome coronavirus (MERS-CoV) was shown to occur via DNA methylation^4^. DNA methylation changes at cytosine-phosphate-guanine (CpG) sites have been increasingly leveraged in the emerging field of clinical epigenetics to characterize unique epigenetic signatures that diagnose disease. To date, considerable success has been demonstrated in developing highly accurate and robust machine learning (ML)-based disease classifiers using DNA methylation patterns to differentiate Mendelian disorders^17^, behavior disorders^18^, coronary artery disease^19^, and some cancers^20–22^. Consequently integration of a methylation-based disease classification can result in relevant improvement in clinical practice^23,24^.

With a goal to leverage Illumina’s Infinium MethylationEPIC Array to classify differential methylation signatures of SARS-CoV-2-positive (hereafter referred to as SARS-CoV-2+, regardless of additional symptoms) and control peripheral blood DNA samples (either confirmed SARS-CoV-2 negative or samples collected prior to the SARS-CoV-2 pandemic), we conducted this study to determine whether DNA methylation patterns could differentiate SARS-CoV-2-infected patients from non-infected patients from whole blood obtained from patients. Our secondary objective was to determine whether DNA methylation patterns could differentiate patients with SARS-CoV-2 infection who go on to develop severe disease. In this study, we identified a strong COVID-19-specific epigenetic signature in peripheral blood driven by key immune-related pathways related to SARS-CoV-2 infection status, disease severity, and clinical deterioration.

## Methods

### Source of data

This protocol was reviewed and approved by the Colorado Multiple Institutional Review Board (COMIRB) and the research adheres to the ethical principles of research outlined in the U.S. Federal Policy for the Protection of Human Subjects. SARS-CoV-2+ were defined as those patients who tested positive for SARS-CoV-2 infection via a routine diagnostic RT-PCR assay in the Biobank at the Colorado Center for Personalized Medicine (Thermo Fisher Scientific, Waltham, MA) or in the UCHealth University of Colorado Hospital Clinical Laboratory (Roche Diagnostics, Indianapolis, IN) of a nasopharyngeal swab collected in viral transport media; controls were defined as those who tested negative. Peripheral blood DNA samples were collected in EDTA tubes from patients seen at the UCHealth University of Colorado Hospital and tested for SARS-CoV-2 epigenetic signatures starting on March 1, 2020. Blood specimens were collected from patients consented to the University of Colorado COVID-19 Biorepository (https://research.cuanschutz.edu/university-research/covid-19-clinical-research/covid-19-biobank-specimen-repository) or the University of Colorado Emergency Medicine Specimen Bank (EMSB)^25^. Control subjects included patients from each study who tested negative for SARS-CoV-2 infection during the index visit. Through the University of Colorado COVID-19 Biorepository and the EMSB, patients tested were consented for blood collection and data abstraction from their electronic health record (EHR). Data obtained from EHR abstraction included demographics, past medical history, laboratory testing (including SARS-CoV-2), treatments, vital signs, hospital disposition, and clinical outcomes. In addition, previously collected samples from patients with acute upper respiratory viral infections (SARS-CoV-2 negative/pan-negative for upper respiratory viral infections/positive for non-SARS-CoV-2 upper respiratory viral infections) between February 5, 2018 and January 1, 2020 were obtained through the EMSB as SARS-CoV-2-negative controls. Additional biospecimens included discarded clinical samples from patients not approached for biorepository enrollment through the UCHealth University of Colorado Hospital Clinical Laboratory. Discarded samples were linked to a limited EHR dataset through the Colorado Center for Personalized Medicine’s health data warehouse, Health Data Compass, and then deidentified. The limited dataset included age, gender, race, ethnicity, viral test status (SARS-CoV-2 and other upper respiratory viruses), and clinical outcomes. The use of discarded samples and accompanying limited datasets was determined to be exempt from Institutional Review Board approval and the need for informed consent by COMIRB. All samples were frozen at −20 °C after collection prior to processing for methylation analyses.

### Customization of the Infinium MethylationEPIC Array

Following a literature review of known epigenetic associations with respiratory viral infections from recent CoV outbreaks, we selected additional content to enrich Illumina’s Infinium MethylationEPIC Array^26^. We specifically enriched for known HLA alleles accounting for known genomic variation^27^ as well as multiple alternative haplotypes and unpublished reference sequences spanning the major histocompatibility complex genomic region, the natural killer cell immunoreceptor, and other immunogenetic loci (e.g., cytokines, interferon response genes), to enhance the sensitivity of immune response detection. The custom panel targeted 262 genes with 7831 additional probes. While the majority of the additional probes targeted unique sequences within the genome, a number of probes were intentionally designed to target genomic sequences with a limited degree of repetitiveness. The list of genes and the Illumina IDs for the probes that target these genes are given in Supplementary Data 1.

### Methylation array and quality assessment

#### DNA extraction

Biospecimens were accessioned and tracked via the Colorado Anschutz Research Genetics Organization (CARGO) laboratory information management system (LIMS). Genomic DNA was extracted from SARS-CoV-2+ peripheral blood on the bead-based, automated extraction Maxwell(R) RSC System (Promega) in a biological safety cabinet in compliance with CDC safety guidelines and procedures for handling SARS-CoV-2 biospecimens (biospecimens from SARS-CoV-2+ cases) and from controls on the Autogen FlexSTAR+ using the Autogen’s FlexiGene Blood Extraction Kit (Holliston, MA). All DNA samples were quantified using both absorbance (NanoDrop 2000; Thermo Fisher Scientific, Waltham, MA) and fluorescence-based methods (Qubit; Thermo Fisher Scientific, Waltham, MA) using standard dyes selective for double-stranded DNA, minimizing the effects of contaminants that affect the quantitation. DNA quality was assessed using an Agilent TapeStation (Agilent, Santa Clara, CA). Samples were then uploaded to CARGO’s LIMS, barcoded, and labeled.

### Bisulfite conversion and amplification

Purified DNA samples were processed using the Zymo EZ-96 DNA Methylation bisulfite conversion kits (Zymo, Irvine, CA) as described previously^28^. The product of this process contains cytosine converted to uracil if it was previously unmethylated. The bisulfite-treated DNA was subjected to whole-genome amplification via random hexamer priming and Phi29 DNA polymerase, and the amplification products were then enzymatically fragmented, purified from dNTPs, primers, and enzymes, and applied to the Illumina chip as described elsewhere^29^.

### Hybridization and single-base extension

The bisulfite-converted amplified DNA products were denatured into single strands and hybridized to the customized Infinium 850K BeadChip (EPIC+; Illumina Inc., San Diego, CA) via allele-specific annealing to either the methylation-specific probe or the non-methylation probe. Hybridization to the chip was followed by single-base extension with labeled di-deoxynucleotides according to Illumina’s Infinium protocol at the CARGO laboratory^28^.

### Fluorescence staining and scanning of chip

The hybridized BeadChips were stained, washed, and scanned to show the intensities of the un-methylated and methylated bead types using Illumina’s iScan System.

### Data processing and quality control (QC)

IDAT files were processed, filtered, and normalized using the SeSAMe R package^30^. Type I probe channel was empirically determined from signal intensities. Probe detection *P* values (representing the ability to differentiate true signal from background fluorescence) were calculated for each color channel using pOOBAH, which leverages the fluorescence of out-of-band (OOB) probes. Normalization was performed using noob, which uses OOB probes to perform a normal-exponential deconvolution of fluorescent intensities^31^. Finally, a common dye bias that results in greater intensities in the red color channel was corrected to ensure that the distribution of intensities in the two color channels were equal. Probes with detection *P* values >0.05 were removed, as well as probes overlapping single-nucleotide polymorphisms with global minor allele frequency >1% in dbSNP, probes with poor mapping, and probes containing non-unique sequence according to Zhou et al.^32^. Beta values were logit-transformed into *M* values for modeling. Probes with >25% missingness were removed. Remaining missing values were then imputed with mean probe *M* value.

### Selection of discovery/training and testing cohorts and controls

**Fig. 1:**
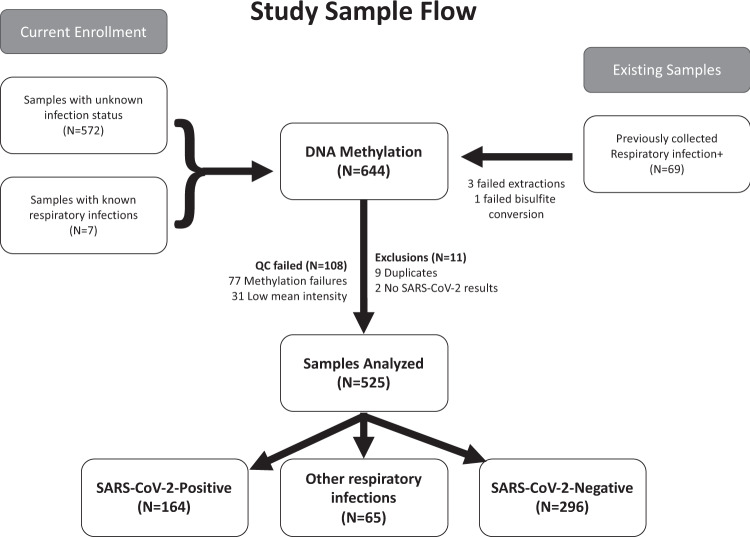
Flowchart of the study sample collection. Six hundred and forty-eight samples were collected for analysis, of which 644 were processed on MethylationEPIC arrays. Five hundred and twenty-five arrays passed quality control and were included in the final analysis.

Case–control analyses were performed using the entire genotyped dataset passing epigenetics QC, with SARS-CoV-2 infection status determined as described above (see Fig. 1 for a summary of the workflow). Analyses were repeated including and excluding controls with other upper respiratory infections validated by clinical respiratory panels. Measurements of disease severity and progression (e.g., hospitalization, ICU admittance, ventilator use) were extracted from chart review within the UCHealth EHR.

### Control for batch effect and robustness of the identified epigenetic signatures

To minimize possible batch effects and other sources of variability, samples were split into SARS-CoV-2+ and SARS-CoV-2-negative control sets, randomized within sets to account for unavailable phenotypes, and then distributed across chips. To reduce batch and plating effects a minimum of two SARS-CoV-2+ and two SARS-CoV-2-negative control samples were run on each chip (12 chips per plate, 8 samples each) and positive/negative status was randomized across the chip.

### Epigenome-wide association study (EWAS) with COVID-19 disease status

Preprocessing was performed using the GLINT^33^ package for association testing and estimating components to adjust for population structure (EPISTRUCTURE^34^) and we used ReFACTor^35^ to account for cell-type proportions. We chose ReFACTor to account for cell proportion information in a data-driven fashion. The linear mixed-effects model in GLINT was fit to each probe, testing for differences based on COVID-19 disease status while correcting for age, sex, chip position, 6 ReFACTor components, 1 EPISTRUCTURE component, and a variance component representing individual covariance^36^. Enrichment of top hits in common databases was performed using enrichR^37^. Probes were sorted by adjusted *P* value and the top 800 genes to which differentially methylated probes map were used as input to perform overrepresentation enrichment analysis within Gene Ontology (GO) categories, Kyoto Encyclopedia of Genes and Genomes pathways (KEGG), BioPlanet, and WikiPathways^38–41^. Probes were annotated to CpG island and genic regions using annotatr^42^.

### Clinical outcome stratification

Clinical data were abstracted via detailed chart review for all EMSB patients. COVID-19 disease severity was determined by an ordered severity score of (1) discharged from emergency department; (2) admitted to inpatient care; (3) progressed to ICU; and (4) death. We also determined a hospital duration variable, where individuals without a measured hospital stay (i.e., discharged from the emergency department) were assigned 0 and individuals who died were removed from the cohort for length of stay analysis to minimize bias associated with timing of decisions to withdraw care.

### Construction and validation of a prediction model

Predictive modeling was performed using the Lasso^43^ and Elastic Net^44^ algorithms for sparse penalized regression modeling available in the *glmnet* software package^45^. For each prediction model, only autosomal methylation probes passing QC were included, to remove potential confounding from sex-linked chromosomes. No demographic, clinical, or cell count variables were included in the predictive models, requiring the algorithm to pick CpG sites with strong enough associations to surpass the level of penalization of the hyperparameters across the entire least angle regression path. For each trait of interest, a separate model was created and best-fitting parameters were chosen after tenfold cross-validation either by maximizing area under the receiver-operator characteristic curve (AUC for dichotomous traits) or minimizing mean-squared error (MSE for quantitative traits). Each was fit across a grid of parameters representing various strengths of penalization and combination of L1 and L2 penalties under the weighted elastic net model. Both the days of hospitalization and case severity were modeled as continuous outcomes. To assess performance for quantitative traits in a manner comparable to dichotomous traits, we swept across potential cutpoints to estimate AUCs for this newly derived dichotomous variable. While case–control status was the primary phenotype of interest, measures of severity were assessed in SARS-CoV-2+ cases only.

To estimate stability of estimation in parameters, we performed 100 iterations of model training and testing. Within each iteration for case–control and severity outcomes, we employed tenfold cross-validation to derive the model and a held-out set of 30% removed from train/test to gauge out-of-sample performance of the best-fitting model. Our train/test and validation splits were created within each stratum to preserve representation across all outcomes and reflect the distribution across the total dataset. For hospitalization duration, the train/test/validation models had instability in convergence and so we reverted to a train/test model using the tenfold cross-validation within the default cv.glmnet() function. We assessed overall performance for the dichotomous COVID+/COVID− case–control status using out-of-sample AUC, the F1 score (a measure of the relationship between precision and recall), the distribution of best-fit *λ* penalty via cross-validation, and the number of probes chosen in the final model. For the quantitative outcomes, we assessed overall performance using out-of-sample *R*^2^, the slope of the model, and *λ* number of probes. Finally, these were stratified each across the elastic net weights (*α*) from 0.01 to 1, representing the proportion of ridge (L2) vs Lasso (L1) penalty to choose a final model. All models included nonzero *λ* to encourage sparsity (a L2-only model would include prediction from the entire array). Final models described in results were chosen based on best-performing (maximum *R*^2^ or AUC) vs median values for each chosen set of hyperparameters. The final, out-of-sample best-fit prediction for each outcome was considered the “methylation score” used in downstream modeling, characterization of association, and determination of potential confounding with demographic and blood cell proportion characteristics.

### Reporting summary

Further information on research design is available in the Nature Research Reporting Summary linked to this article.

## Results

### Study cohort

We identified 675 patients tested for either SARS-CoV-2 or other acute upper respiratory infections. Of these, 164 were SARS-CoV-2+ by RT-PCR, 58 historical EMSB patients had positive (non-SARS-CoV-2) acute upper respiratory viral RT-PCR tests, 7 had positive (non-SARS-CoV-2) acute upper respiratory viral RT-PCR tests during the pandemic, and 296 were negative for all viral infections and thus served as controls. We excluded 32 samples from the dataset as these were derived from a run with failed hybridization and removed 8 duplicates, resulting in a final cohort of 525 (Fig. 1). Supplementary Table 1 summarizes the demographics and clinical outcomes of patients tested, including proportion of patients with other acute upper respiratory infections. Incidences of non-SARS-CoV-2 respiratory infections are displayed in Supplementary Table 2. The median time from sample collection to hospital admission was 0 days (interquartile range (IQR): 0, 1). In all, 83.4% of samples were collected on the day of admission and only 8.7% were collected >5 days after hospital admission. Samples from patients who were SARS-CoV-2 positive were drawn with the first blood sample in the emergency department 83% (median blood draw: 0 days, IQR: 0, 1 days) of the time; other samples drawn later in the hospital admission in this group were from patients who developed COVID while admitted to the hospital. Samples from two SARS-CoV-2-positive patients were obtained 6 and 9 days prior to hospital admission. Samples from SARS-CoV-2-negative patients were drawn with the first blood sample in the emergency department 80% (median blood draw: 0 days, IQR: 0, 2 days) of the time and 95% were drawn within 7 days of hospital admission. No samples were obtained on days before hospital admission in the SARS-CoV-2-negative patients.

### Disease-specific DNA methylation signature and differentially methylated probes

**Fig. 2:**
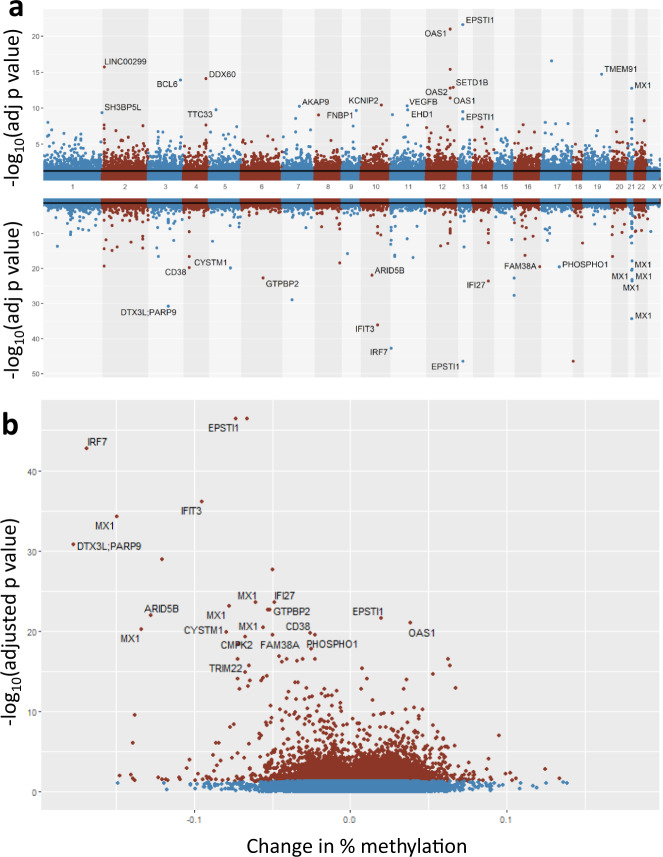
Differentially methylated CpGs associated with SARS-CoV-2 infection. **a** Miami plot (top panel) of hypermethylated (top) and hypomethylated (bottom) probes in SARS-CoV-2+ compared to control samples. Significance lines represent FDR-adjusted *P* value <0.05 threshold. **b** Volcano plot of significant (red; FDR-adjusted *P* value <0.05) CpG sites (blue CpG sites have FDR-adjusted *P* value >0.05). Change in percentage methylation on the *x* axis represents the difference in average beta value at a site between cases and controls. Probes for intergenic CpG sites do not have gene annotations. Data used to plot this figure are available as Supplementary Data 5.

We first performed an EWAS to identify biological signals associated with COVID-19 disease status. After adjustment for age, sex, array position (batch effect), cell proportions via ReFACTor and ancestry via EPISTRUCTURE components, EWAS of COVID-19 disease status in 164 SARS-CoV-2+ compared to 296 controls yielded 13,033 significant CpGs mapping to 6117 unique genes at false discovery rate (FDR)-adjusted *P* value < 0.05 (Fig. 2 and Supplementary Data 2), with moderate inflation that is typical of EWAS^46^ (Supplementary Fig. 1). In total, we observed 35 probes with an unadjusted *P* value < 10^−20^, and 183 with an unadjusted *P* value < 10^−10^. Significant probes overlap 1625 CpG islands and 1001 FANTOM5^47^ enhancers (Supplementary Fig. 2). We observed that 52.1% of all significant probes are hypermethylated; however, 78% of the top 100 probes sorted by adjusted *P* value are hypomethylated (Fisher’s Exact Test *P* value = 9.46 × 10^−8^). Custom probes on the EPIC+ chip are enriched in significant EWAS results (*P* value = 9.94 × 10^−7^, Fisher’s Exact Test): specifically, 1.72% of EPIC probes are significant as opposed to 2.51% of custom probes. Principal component analysis of top associations reveals clustering by COVID-19 disease status (Supplementary Fig. 3). Because of concerns that population admixture may confound results, the COVID-19 disease status EWAS was repeated with EHR-defined race and ethnicity as additional covariates beyond that modeled via EPISTRUCTURE and mixed-effects modeling. This had a minimal effect on results.

Top hypomethylated CpG sites show strong enrichment for interferon and viral response-related pathways including Type I Interferon Signaling Pathway (KEGG, adjusted *P* value = 7.40 × 10^−10^) and Negative Regulation of Viral Genome Replication (GO:BP, adjusted *P* value = 1.93 × 10^−6^; Supplementary Fig. 4a). Hypermethylated CpG sites also show enrichment for relevant biological processes such as Focal Adhesion (GO:CC, adjusted *P* value = 0.0187; Supplementary Fig. 4b). cg17114584, the third most significant probe with an adjusted *P* value of 1.78 × 10^−43^, shows 16.9% hypomethylation in cases. This CpG is located in exon 6 of the interferon regulatory factor 7 (*IRF7*). *IRF7* encodes a transcription factor that regulates the expression of interferon a and b, as well as interferon-stimulated genes. Other top CpGs are in genes relevant to viral response: *OAS1* (2’-5’-oligoadenylate synthetase 1) is interferon-induced and activates RNase L, which degrades viral (and cellular) RNA (adjusted *P* value 1.05 × 10^−21^, 3.8% methylation change). *MX1* encodes an interferon-induced GTPase that inhibits viral replication. *DTX3L* and *PARP9* form a complex that is involved in interferon-mediated antiviral defenses. This complex has also been shown to promote M1 polarization in macrophages by preventing STAT1 phosphorylation^48^. *IFIT3* encodes another interferon-induced antiviral protein. Overall, we observe strong hypomethylation of interferon- and viral response-related pathways, which is expected as these pathways are activated transcriptionally in SARS-CoV-2+ individuals^49^.

### Specificity of the COVID-19 disease signature from other respiratory infections

**Fig. 3:**
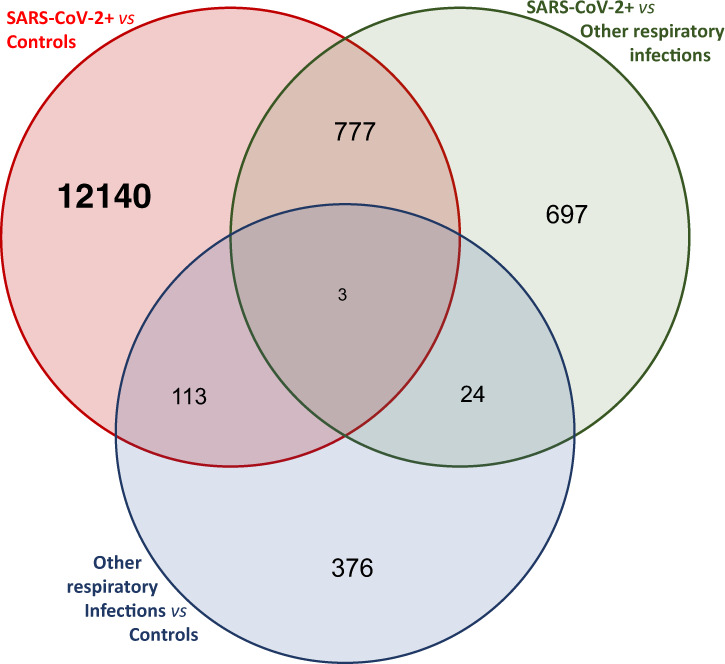
Overlap of differentially methylated CpGs between disease groups. Venn diagram of overlaps between SARS-CoV-2+–Control EWAS (13,033 significant probes), SARS-CoV-2+–other respiratory infection EWAS (516 significant probes), and other respiratory infection–Control EWAS (1501 significant probes).

We next compared 164 SARS-CoV-2+ samples to 65 samples with other upper respiratory infections to determine the specificity of the methylation signature to SARS-CoV-2. This analysis yielded 1501 significant CpGs (adjusted *P* value < 0.05) (Supplementary Data 3), of which 780 (52%) were present in the SARS-CoV-2+ compared to controls analysis (Fig. 3). Comparison of 65 other (non-SARS-CoV-2) upper respiratory infection samples to controls yielded 516 significant CpGs (Supplementary Data 4), of which 116 (22%) were present in the SARS-CoV-2+ compared to controls analysis. Furthermore, examination of the strength of the signal demonstrates that the shared probes in the SARS-CoV-2+ vs control and SARS-CoV-2+ vs other upper respiratory infections analysis have low *P* values and high effect sizes, whereas this is not the case for probes shared by SARS-CoV-2+ vs control and other upper respiratory infections vs control analyses (Supplementary Fig. 5a). These comparisons suggest high specificity of the COVID-19 disease epigenetic signature. To further investigate this, we examined the significant CpGs from our COVID-19 disease signature compared to control EWAS. We observe the same trend of high correlation of effect sizes (methylation change) in SARS-CoV-2+ compared to control and SARS-CoV-2+ compared to other respiratory infections (Pearson *R* = 0.87; *P* < 2.2 × 10^−16^) and very low correlations of effect sizes in SARS-CoV-2+ compared to control and other upper respiratory infections compared to control analyses (Pearson *R* = −0.027; *P* = 0.0022) (Supplementary Fig. 5b). While we do not have sufficient power to examine the specific viruses (other CoV, influenza, etc.), these results strongly point to the specificity of our COVID-19 disease epigenetic signature to detect SARS-CoV-2 infection.

### Development and validation of a classification model for prediction of disease classes and disease severity

**Fig. 4:**
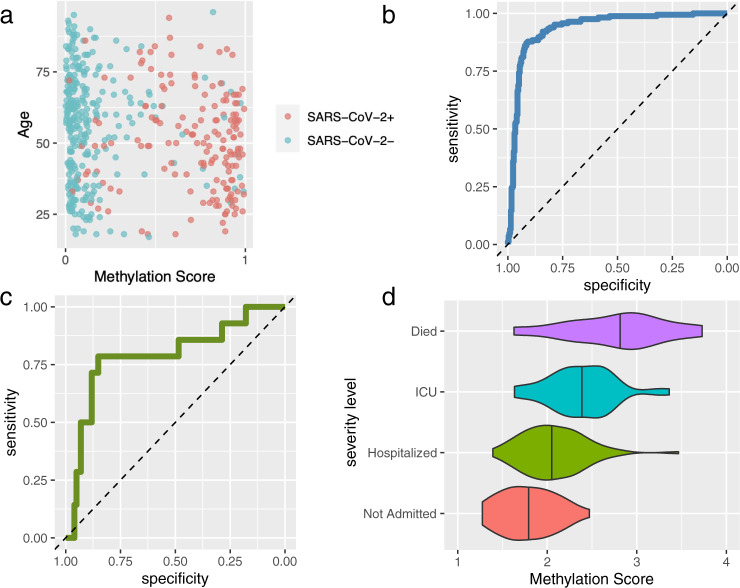
Performance of SARS-CoV-2 infection status and severity predictive models. **a** Out-of-sample case–control methylation score for all 460 individuals (164 SARS-CoV-2+, 296 SARS-CoV-2−) compared to case–control status, plotted by biological age. **b** Receiver-operating characteristic (ROC) curve for data in **a**. **c** ROC curve of cross-validated prediction of long hospital duration. **d** Violin and jittered scatter plots of severity methylation scores for each outcome in cases. Data used to plot this figure are available as Supplementary Data 6.

To combine methylation data across the genome into a single predictor, we employed ML models of sparse regression trained via cross-validated *glmnet*^45^ as described in “Methods.” To determine the sensitivity of our model, 460 subjects (SARS-CoV-2+ vs controls) from the testing cohort were supplied to the classification model, with prediction optimized after the approach defined in “Methods.” Only methylation probes were used in feature selection. All models showed relative stability across iterations (Supplementary Fig. 6) and yielded sparse results. Details of each top model are available in Supplementary Table 3. The best-fitting model has a performance of 93.6% in cross-validation for detecting SARS-CoV-2 infection (Fig. 4a, b). Model performance was similar in females and males (93.7 and 93.5%, respectively). In addition, model performance on older individuals and younger individuals (median age = 56 years) was comparable: 94.4 and 92.8%, respectively. Similarly, race/ethnicity information was not significantly correlated with case–control score (all groups *P* > 0.05). When age and race/ethnicity categories were included in a multivariable model along with our prediction score, no additional covariates significantly predicted COVID-19 disease status (all other *P* > 0.4). Similarly, BMI was not associated (*P* ~ 0.4).

To determine the direct association of methylation with clinical outcomes, an additional logistic regression was performed for the subset of individuals with complete blood cell count (CBC) data (341 individuals total). The inclusion of additional blood cell count data did not impact the association between the methylation score and outcome (*P* value < 2 × 10^−16^ with or without adjustment), and in the larger CBC model (including total hematocrit, white blood cell count, platelets, neutrophils, lymphocytes, monocytes, eosinophils, and basophils), only hematocrit (*P* ~ 0.05) approached nominal significance. The inclusion of hematocrit moderately improved Akaike information criterion in logistic regression but with limited performance increase in multivariable modeling AUC (93.6 vs 94.1%).

Severity analysis focused on hospital length of stay (median duration: 6 days, IQR 3–11, max 53 days), as well as across the spectrum of severity (34 discharged from emergency room, 84 hospitalized, 35 admitted to ICU, and 11 deaths). The best-fitting model for hospital duration had a cutpoint at 20 days, yielding an AUC of 79.6% with 14 individuals with longer stays vs 135 with shorter stays (or 0 days in hospital) (Fig. 4c). Dichotomizing the best-fit severity measurements yields AUCs of 79.1, 80.8, and 84.4 for hospital admission vs discharge, floor hospital admission vs ICU, and survival vs death, respectively (Fig. 4d).

## Discussion

Here we report DNA methylation profiling in conjunction with analysis using ML techniques to identify a SARS-CoV-2-specific epigenetic signature in peripheral blood from a large cohort of individuals tested using conventional RT-PCR technology. We also describe the development of a classification algorithm that has high sensitivity and specificity in predicting infection and in-hospital clinical deterioration and that confidently rejects the probability of healthy individuals to be affected by SARS-CoV-2 infection. While any predictive signal invites concern of potential confounding, the methylation signature (derived solely from CpGs, not including any clinical or demographic information) we observe is not driven by confounding either from demographics or typical laboratory measurements (e.g., blood cell counts, BMI). Our findings suggest that measurement of methylation signals that arise during and after SARS-CoV-2 infection may provide clinicians the ability to detect viral infection as well as predict patient clinical course after viral challenge. Unlike sequencing, RT-PCR, and antibody tests, the methylation array is able to predict the severity of SARS-CoV-2 infection and ultimately could provide clinicians with information on how to manage patients infected with SARS-CoV-2.

Our results support the hypothesis that the host epigenome, as measured in peripheral blood, is modified by infection from SARS-CoV-2 and can be used to identify novel biology and it is useful for clinical diagnosis, prognosis, and triage. Despite being a heterogeneous tissue, we relied on peripheral blood as the target tissue because it has proven to be a reliable source for generating epigenetic signatures and disease classifiers in other settings^50–56^. In this study, we observed many methylation changes that are, on average, >10% differentially methylated in the SARS-CoV-2+ group, including *IRF7* and *MX1* interferon-related genes. These are much larger effect sizes than typically observed in EWAS in peripheral blood^57^ and similar to the clinical utility of epigenetics observed in cancer^22^. We did not observe confounding by cell proportions, measured by CBC from the EHR, providing strong support for the epigenetic signature of SARS-CoV-2. Although cell-type heterogeneity can be a strong confounder in epigenetic studies^58–60^, we did not pursue adjustment for cell proportions beyond adjustment for cell-type proportions using ReFACTor^35^ because our primary objective is to develop a COVID-19 disease-specific diagnostic methylation platform, rather than interrogate the underlying pathology.

To validate the customized EPIC methylation platform as a reliable tool for the clinical diagnosis of COVID-19 disease, we performed an EWAS with SARS-CoV-2 infection status. We observed that the epigenetic signature of SARS-CoV-2 infection is enriched for pathways related to host viral response, and specifically for Type I Interferon signaling that is a hallmark of host response to this virus^61^. Our findings of altered DNA methylation in interferon response genes are in concordance with published results of changes in the expression of interferon response genes by SARS-CoV and MERS-CoV viruses through changes in histone modifications^2,3^. One of the most significant probes (adjusted *P* = 1.77 × 10^−43^, 16.9% hypomethylation) is located in the gene encoding *IRF7*; loss-of-function variants in 13 genes including *IRF7* were recently found to be associated with life-threatening COVID-19-associated pneumonia^62^. Another interferon-induced gene, *OAS1*, was similarly significant (adjusted *P* value 1.05 × 10^−21^, 3.8% methylation change). In a recent GWAS on critical illness due to SARS-CoV-2, significant associations and replication were observed for variants in the OAS gene cluster, which includes *OAS1*^63^, for which variants had previously been associated in candidate gene studies of SARS-CoV infection^61,64^. Also, in a Mendelian randomization study it was recently shown that increased circulating OAS1 proteins were associated with reduced SARS-CoV-2 susceptibility and disease severity^65^. Collectively, published genomics studies support several of the strongest associations observed in our study.

Previous work also demonstrated that viruses that cause severe disease (e.g., MERS-CoV, H5N1) alter host response by changing methylation landscape of antigen-presenting genes in the HLA region^4^. While we did not observe genome-wide significant signals at classical HLA alleles, we observed six FDR *q* < 0.05 probes in the region, in *HLA-V*, *DOA*, *DQA1*, *DQA2*, and *DRA*, albeit with attenuated significance compared to top CpGs (minimum *q* ~ 0.0109), suggesting that the mechanism of host manipulation by SARS-CoV-2 may be different. However, these results should be interpreted with caution as interrogation of the HLA region is complex; *HLA-V* for example is a pseudogene^66^.

As the signatures identified in this study appear to be reactive to the disease, aspects of the disease process are expected to impact these results. Namely, we anticipate these changes to be time-sensitive, as the infection will need to have spread enough to induce methylation changes. Similarly, our case–control variables were defined by RT-PCR, which can carry a high false negative rate depending on the stage of infection and timing of sample collection^9^, and may have reduced the classification accuracy. However, we have follow-up EHR information for the patients in this cohort, which minimizes the risk of misclassification bias. We do not expect this potential confounder to affect the measures of severity used in this study as these were determined directly from chart review, but we acknowledge that, for the initial analysis, the numbers of cases may have limited the statistical power and prognostic ability of ML. With additional cases that account for inherent genetic variability within the population, methylation patterns will become more refined and the AUC of these ML models to predict disease severity is likely to increase. While “duration of hospital stay” may not be as immediately actionable as predicting ICU admittance or ventilator use, and it is confounded by pre-existing frailty, social support (or lack of), socio-economic status, and need for ongoing care once the acute illness has receded, the increased variability in the continuous outcome provides improved signal as observed both in our EWAS and our ML modeling. For this analysis, the 11 individuals who died were removed from duration analyses, as their length of stay would be difficult to compare to those who survived. Although the emerging field of epigenetics has demonstrated actionable classification with much smaller sample sizes in contrast to traditional GWAS in other common disease domains^67^, we recognize that additional cases, and in particular understanding the less-severe end of the spectrum (which are likely to be under-reported in data from health systems), will improve our understanding of outcomes across the spectrum of disease severity. We note that, even in our limited sample sizes, the AUCs for ICU admittance still indicate there is signal that can be resolved through future collections. Another limitation of our work is the specificity of the epigenetic signature to SARS-CoV-2 over other respiratory infections. Initial targeted epigenetic analyses demonstrate a trend toward differential methylation, though these findings are limited by low numbers. Currently, we are targeting the collection of biospecimens from patients with respiratory infections other than SARS-CoV-2 for these follow-up studies.

Researchers have previously compared the robustness of DNA methylation profiling vs RNA transcriptome profiling in developing classifiers for different disease states^24,68–70^. One of the advantages of DNA methylation analysis compared to RNA analysis arises from the relative stability of deoxyribonucleic acid over ribonucleic acid^9,71^. The inherent instability of RNA, due to its 2’-OH group and the ubiquitous presence of ribonucleases, requires the use of plasticware, buffers, and processing reagents that are devoid of chemical and enzymatic species that stimulate RNA hydrolysis. Contamination even with a small amount of ribonuclease can degrade RNA samples to the degree where they cannot be analyzed.

The strong signature of viral-driven epigenetic changes may have the ability to detect SARS-CoV-2 infection in patients who never develop symptoms (asymptomatic) and in patients who are not yet symptomatic (pre-symptomatic)^72^. While asymptomatic testing following exposure has increased in recent months, the current testing strategy in the U.S. still predominantly targets symptomatic patients despite estimates that asymptomatic patients represent 40–45% of infected individuals^10,72^. Transmission during the incubation period has been reported, and the viral load of symptomatic and asymptomatic patients is similar^73–76^. The relationship between SARS-CoV-2 viral shedding and risk of transmission is unclear, and the percentage of transmission attributable to asymptomatic or pre-symptomatic infection of SARS-CoV-2 is unknown^77^. We believe that the epigenetics platform may efficiently identify asymptomatic and pre-symptomatic infections, which may, if applied broadly, aid in limiting the spread of SARS-CoV-2.

Due to the widespread occurrence of SARS-CoV-2 and progression to COVID-19 disease, there is the need for scalable testing technologies that can deployed on the national level for surveillance, screening, and prognosis for those infected. The purpose of this study was to identify high-confidence host methylation biomarkers that are able to indicate SARS-CoV-2 infection and predict clinical course of the viral disease in a given patient. This study is a first step toward selecting biomarkers for inclusion on a high-throughput methylation beadchip array specifically for the clinical diagnosis of COVID-19 disease that is also cost-effective given the added value of predicting subsequent clinical outcomes. To that end, we focused on sparse predictive models. Notably, these models are not significantly confounded by demographics or blood cell count information, denoting their specificity to the current infection of the patient, and reducing concern of overfitting to one patient sub-population. These biomarkers can also be used in risk stratification of SARS-CoV-2-infected patients, an unmet need given that none of the existing testing modalities (nucleic acid amplification tests, antigen tests, serology/antibody tests) can achieve this level of specificity. By identifying DNA methylation patterns associated with critical illness, we contend that a methylation test will provide patient-specific treatment targets before critical illness ensues. Pre-emptive dexamethasone^11,78^, anticoagulation^12^, or new pharmacologic targets may prevent mortality, guided by these epigenetics patterns. Although our findings must be complemented with further clinical assessment, our model has shown its capacity to leverage methylation quantification as an innovative strategy to generate epigenetic signatures that assess host response to SARS-CoV-2, which is scalable and may have the ability to confirm positive tests in asymptomatic patients and entire communities, and may ultimately differentially diagnose other viruses causing similar symptoms all within in a comprehensive high-throughput manner.

## Supplementary information

Supplementary Data 1

Supplementary Data 2

Supplementary Data 3

Supplementary Data 4

Supplementary Data 5

Supplementary Data 6

Supplementary Information

Description of Additional Supplementary Files

Reporting Summary

## Data Availability

The datasets generated during the current study are available in the Gene Expression Omnibus repository (accession GSE167202) and include original .idat array files and the final processed data matrix for DNA methylation analyses. Source data used to generate Figs. 2 and 4 are available as Supplementary Data 5 and 6.

## Code availability

Raw array data were processed using SeSAMe 1.7.6 in R 4.0.1. EWAS was carried out using GLINT 1.0.4 on the command line. Machine learning analyses were done using Glmnet v2.0-18 and Data.table v1.11.4 in R 3.5.1. Plotting and consolidation was done in R 4.1.0 using ggplot2 v2_3.3.3 and Data.table v1.14.0. All packages are available through CRAN and Bioconductor.
