# Supplementary material for: Host methylation predicts SARS-CoV-2 infection and clinical outcome
Source: Commun Med (Lond). 2021 Oct 26;1:42. doi: 10.1038/s43856-021-00042-y (PMC8767772; doi:10.1038/s43856-021-00042-y)
Supplement: Supplementary file 7 — Supplementary Information [file 43856_2021_42_MOESM7_ESM.pdf]

Supplementary Information for

Host methylation predicts SARS-CoV-2 infection and clinical outcome

Iain R. Konigsberg[1*], Bret Barnes[2*], Monica Campbell[1], Elizabeth Davidson[1], Yingfei Zhen[1], Olivia Pallisard[1], Meher Boorgula[1], Corey Cox[1], Debmalya Nandy[3], Souvik Seal[3], Kristy Crooks[1], Evan Sticca[1], Genelle F. Harrison[1], Andrew Hopkinson[1], Alexis Vest[1], Cosby G. Arnold[1], Michael G. Kahn[1], David P. Kao[1], Brett R. Peterson[1], Stephen J. Wicks[1], Debashis Ghosh[3], Steve Horvath[4], Wanding Zhou[5], Rasika A. Mathias[1,6], Paul J. Norman[1], Rishi Porecha[2], Ivana V. Yang[1,**], Christopher R. Gignoux[1,**], Andrew A. Monte[1,**], Alem Taye[2,**], and Kathleen C. Barnes[1,**,#]

[1]School of Medicine, University of Colorado Anschutz Medical Campus, Aurora, USA; [2]Illumina, Inc., San Diego, USA; [3]Colorado School of Public Health, University of Colorado Anschutz Medical Campus, Aurora, USA; [4]University of California Los Angeles, Los Angeles, USA; [5]The Children's Hospital of Philadelphia, Philadelphia, USA; [6]Johns Hopkins University, Baltimore, USA

[*]These authors contributed equally
[**]These authors jointly supervised this work
[#]Corresponding author

Supplementary Information

Supplementary Figure 1. Quantile-quantile (QQ) plot of expected vs observed *p*-values in the SARS-CoV-2+ vs. SARS-CoV-2- EWAS. $\lambda = 1.71$.

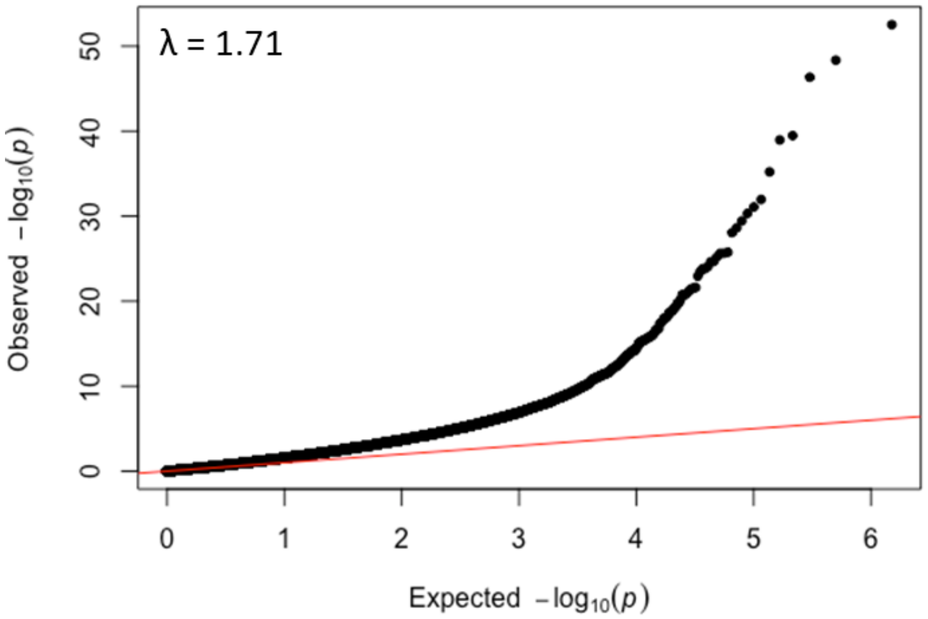

Supplementary Figure 2. Number of **a. CpG island annotations** and **b. genic annotations** overlapping 13,033 significant probes from SARS-CoV-2 infection.

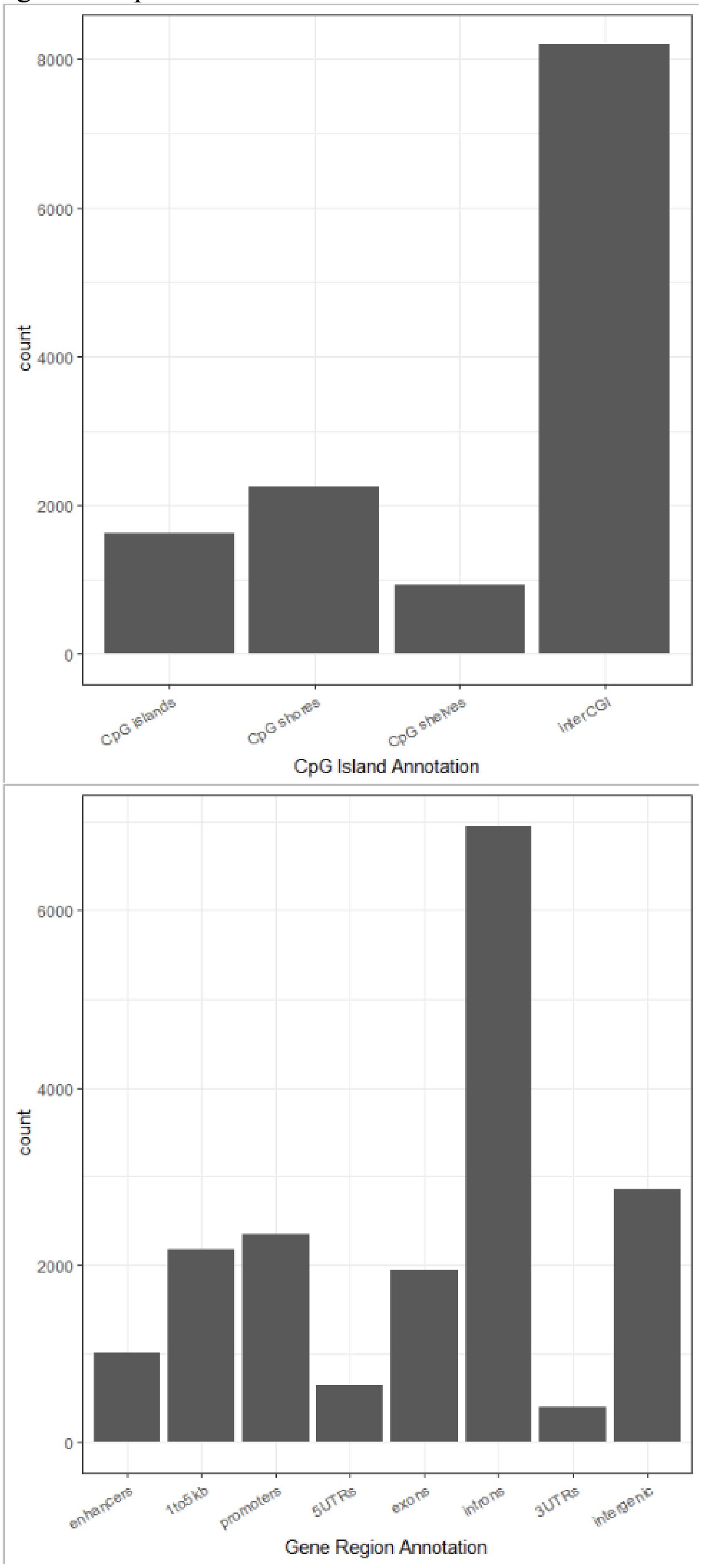

Supplementary Figure 3. Principal component analysis of 13,033 significant CpGs in SARS-CoV-2 disease status EWAS.

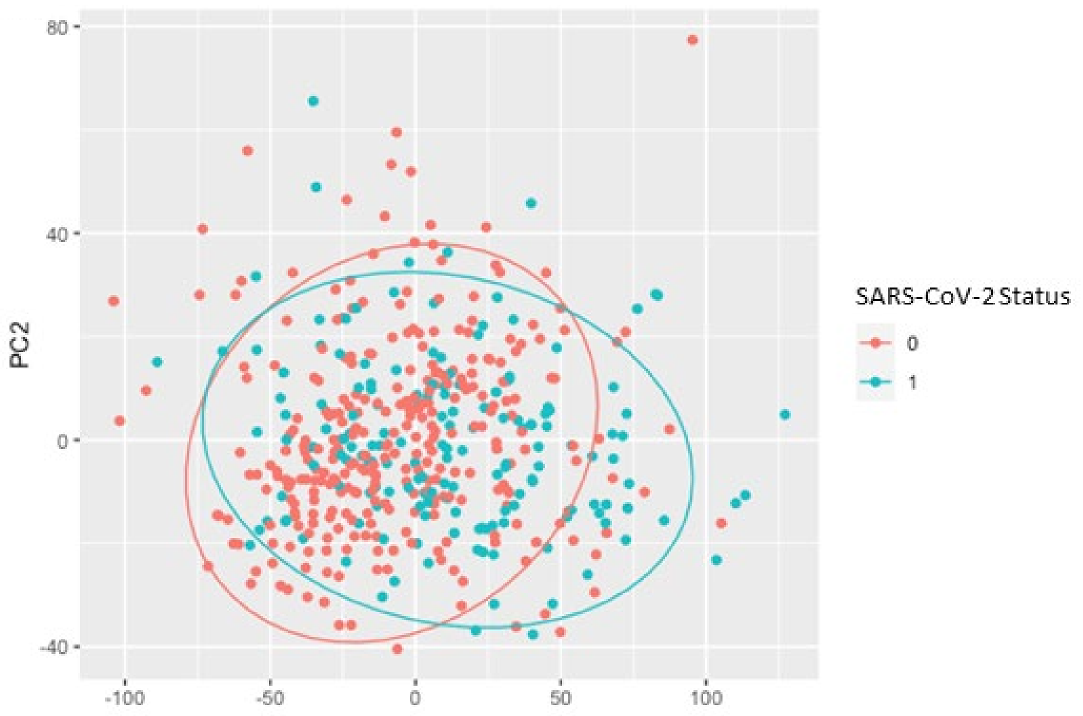

Supplementary Figure 4a. Enriched pathways in top hypomethylated hits from SARS-CoV-2 infection status EWAS.

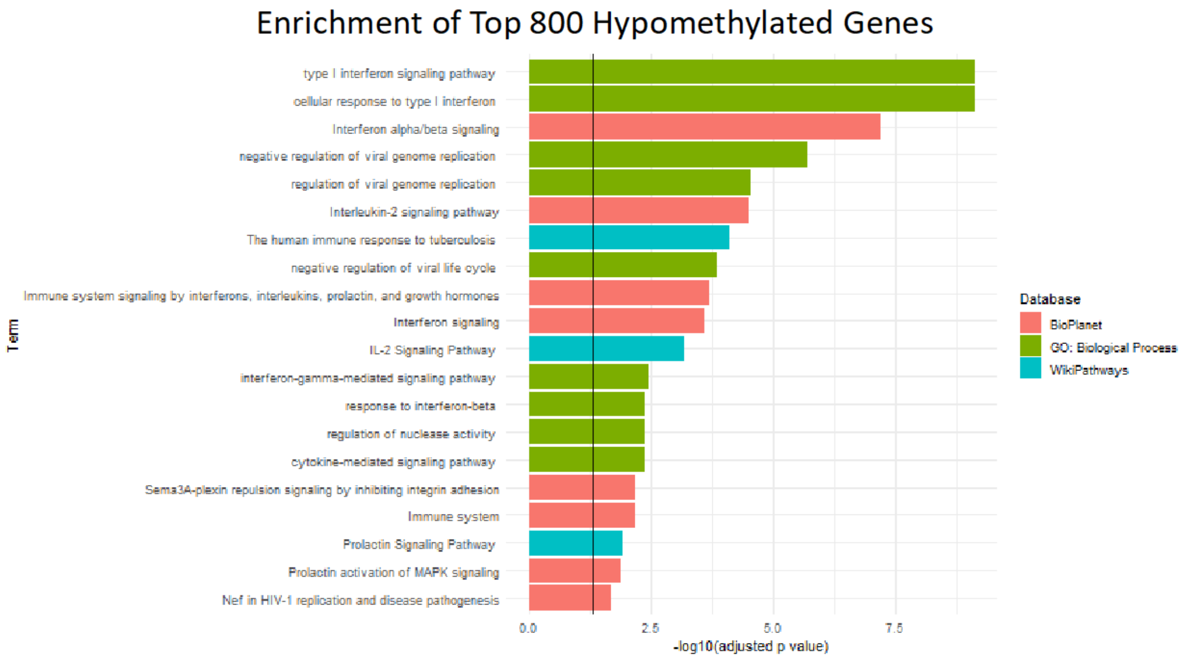

Supplementary Figure 4b. Enriched pathways in top hypermethylated hits from SARS-CoV-2 infection status EWAS.

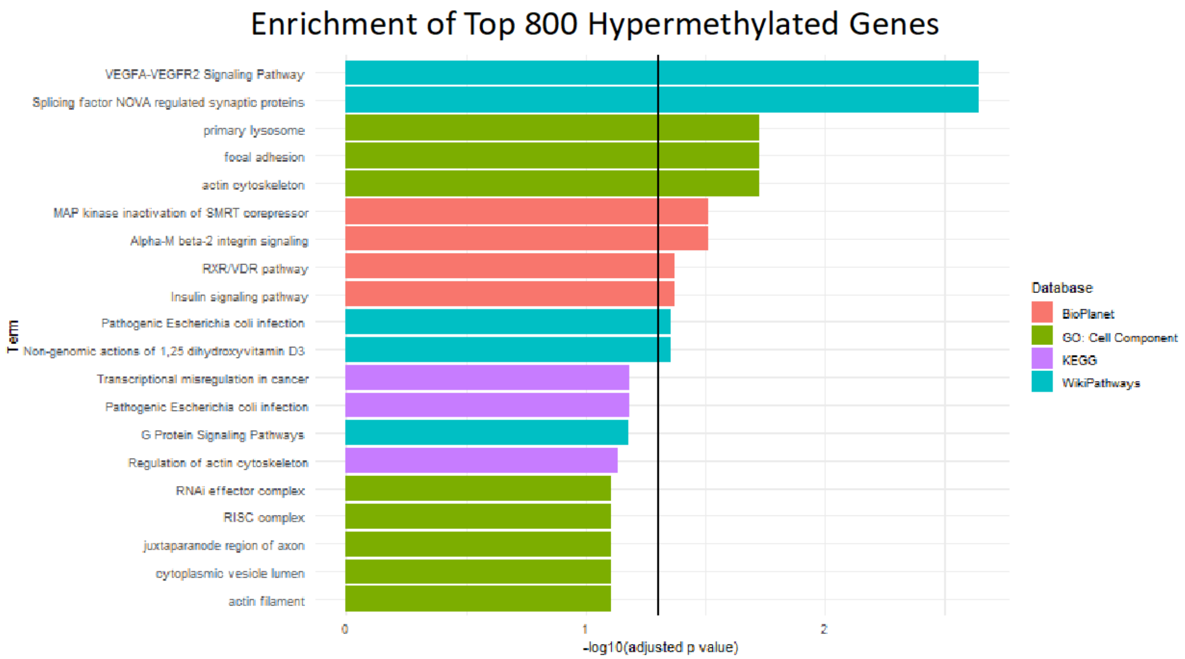

Supplementary Figure 5a. Specificity of the COVID-19 disease EWAS signature. Both volcano plots depict the 13,033 CpGs significant in the EWAS for SARS-Cov-2+ cases compared to controls; the colors are used to represent overlap in those CpGs with EWAS for SARS-Cov-2+ cases compared to other respiratory infections (left panel) and other respiratory viruses compared to controls (right panel). Green dots represent CpGs concordant in directionality between the two EWAS and red dots represent CpGs discordant in directionality between the two EWAS. The black dots are CpGs significant in the SARS-Cov-2+ vs control EWAS but not in the other EWAS.

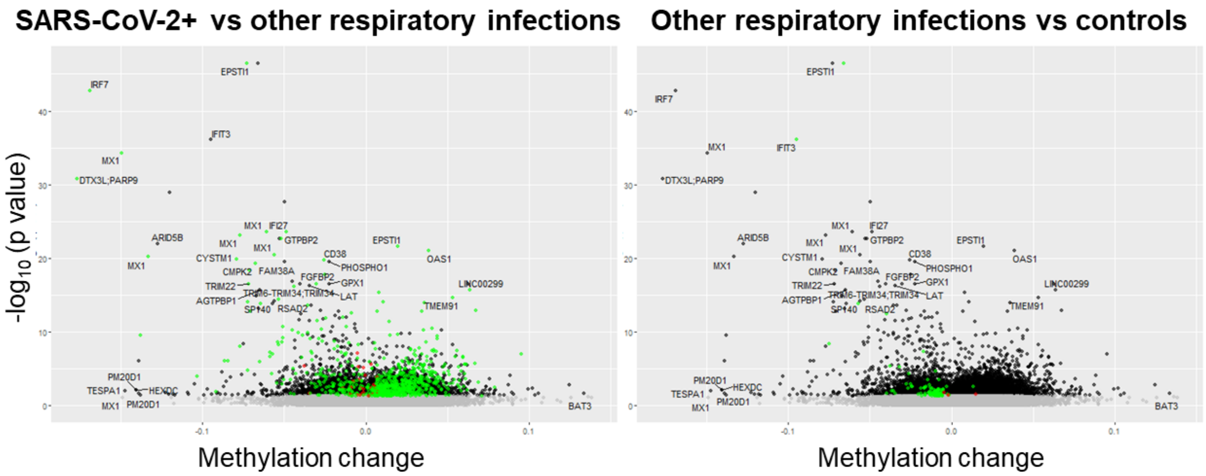

Supplementary Figure 5b. Specificity of the COVID-19 disease EWAS signature. Correlation plots for 13,033 CpGs significant in SARS-Cov-2+ vs control EWAS. Correlations with SARS-Cov-2+ compared to other upper respiratory infections (left panel) and with other upper respiratory infections compared to controls (right panel). Red dots represent CpGs significant in both SARS-Cov-2+ vs control and the other EWAS while gray dots represent CpGs only significant in the SARS-Cov-2+ vs control EWAS.

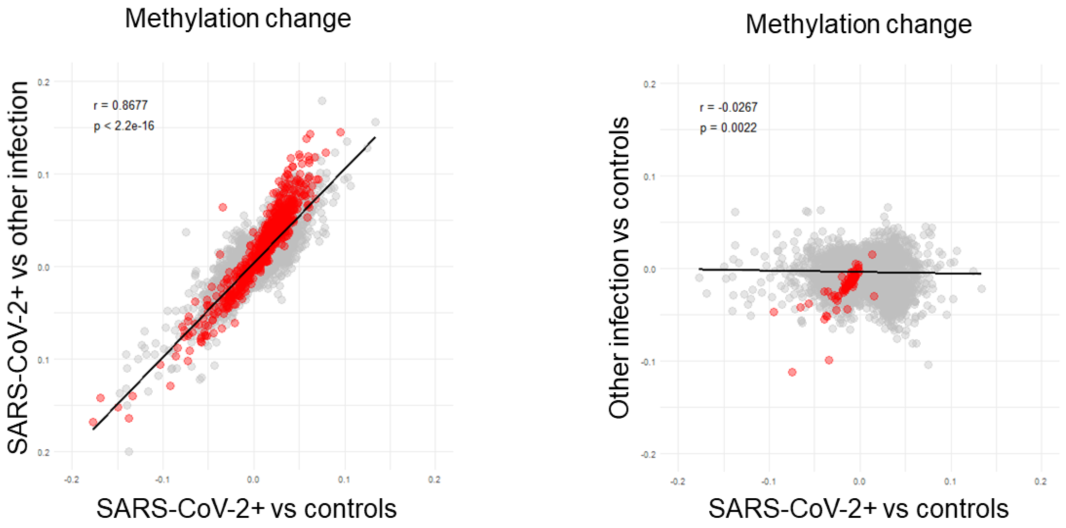

Supplementary Fig 6. Density plots across 100 iterations for each hyperparameter set in glmnet for case-control, hospital duration, and severity. All y-axes reflect density values. For **Ex. Data 6a** as a dichotomous outcome: 1) Number of probes selected in best-fitting model, 2) AUC from out-of-sample model fits across each iteration via Receiver-Operating Characteristic, 3) F1: a statistical relationship between precision and recall, and 4) λ, the best-fitting L1 penalty determined across the least-angle path. For **Ex. Data 6b** and **Ex. Data 6c** as continuous outcomes, we include distributions for number of probes, out-of-sample R^2 predictions, the β value between the predicted score and the true outcome, and the best-fit λ. All plots are stratified by Alpha, the weighted combination of L1 and L2 penalization, where L1 is Lasso regression and L2 is ridge regression. All models smoothed using the default density parameters ggplot2.

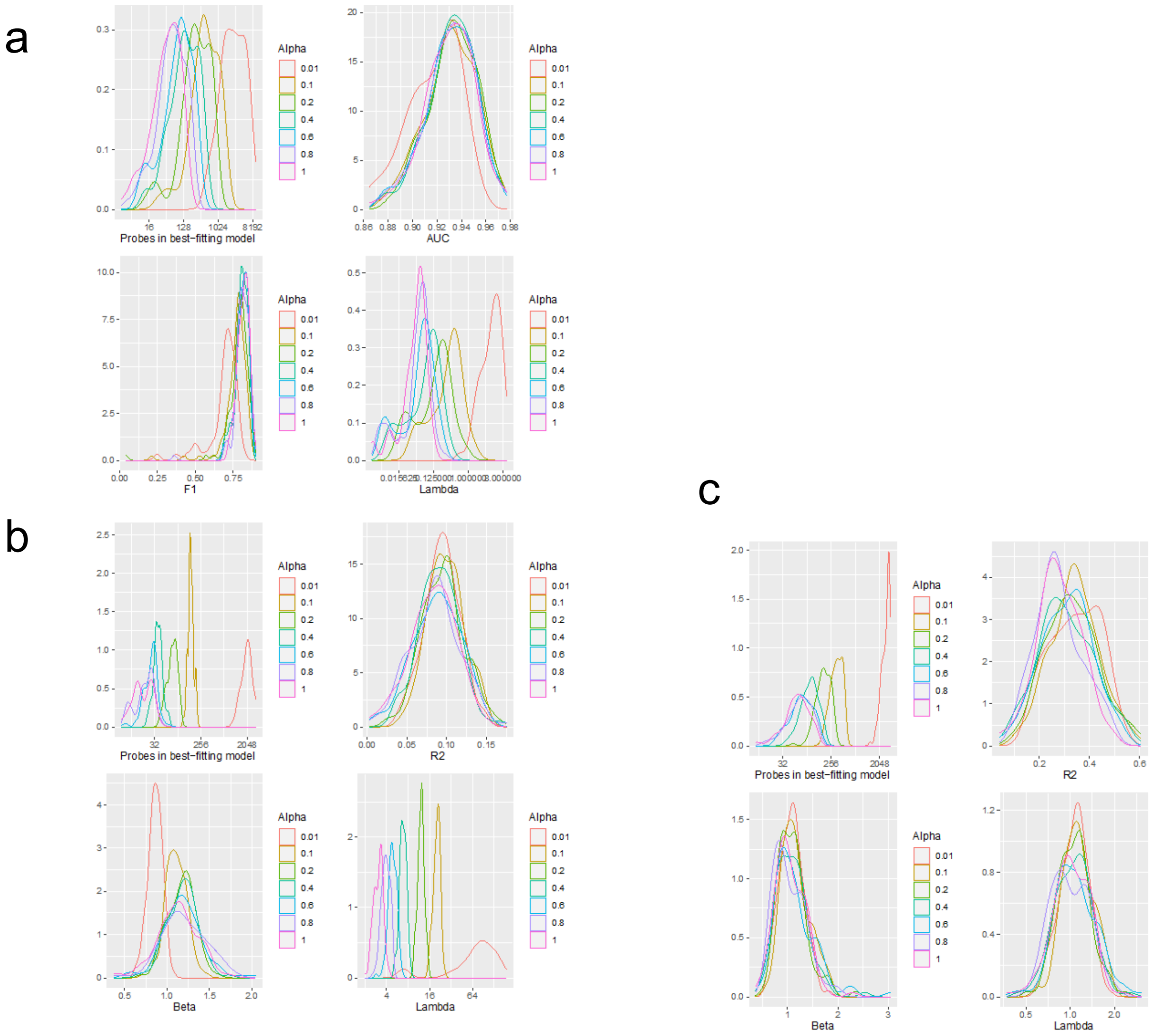

Supplementary Table 1. Demographic summary of all samples analyzed.

Trait	Total Cohort (N=525)	SARS-CoV-2 Positive (N=164)	SARS-CoV-2 Negative (N=296)	Other Respiratory Infection (N=65)
**Age (years)**				
Mean (SD)	54.2 (18.3)	50.5 (17.1)	56.2 (18.9)	54.1 (17.0)
**Gender**				
Female n (%)	251 (47.8)	71 (43.3)	145 (49.0)	35 (53.8)
**Ancestry, n (%)**				
American Indian/Alaskan Native	2 (0.4)	1 (0.6)	1 (0.3)	0 (0)
Asian	18 (3.4)	12 (7.3)	6 (2.0)	0 (0)
Black/African American	92 (17.5)	25 (15.2)	50 (16.9)	17 (26.2)
Native Hawaiian/Other Pacific Islander	3 (0.6)	2 (1.2)	1 (0.3)	0 (0)
White	268 (51.0)	46 (28.0)	184 (62.2)	38 (58.4)
Other	125 (23.8)	70 (42.7)	46 (15.5)	9 (13.8)
Missing	17 (3.2)	8 (4.9)	8 (2.7)	1 (1.5)
**Hispanic or Latino, n (%)**	157 (29.9)	88 (53.7)	57 (19.3)	12 (18.5)
**Body mass index**				
Mean (SD)	30.3 (20.3)	31.6 (10.7)	29.9 (25.2)	29.2 (7.6)
**Emergency Department Disposition, n (%)**				
Discharged	92 (17.5)	33 (20.1)	36 (12.2)	23 (35.4)
Floor admission	346 (65.9)	108 (65.9)	204 (68.9)	34 (52.3)
ICU	82 (15.6)	21 (12.8)	53 (17.9)	8 (12.3)
Death	2 (0.4)	1 (0.6)	1 (0.3)	0 (0)
Ever admitted to the ICU during the hospital stay	126 (24.0)	44 (26.8)	72 (24.3)	10 (15.4)
Ever requiring ventilator during hospital stay	73 (13.9)	27 (16.5)	44 (14.9)	2 (3.1)
Days in hospital				
Median (IQR)	5 (3, 11)	6 (3, 14)	5 (3, 11)	3 (2, 4)

Supplementary Table 2. Respiratory infections identified in non-SARS-CoV-2 positive infections.

**Infectious agent**	**Overall (n=65 patients)**
Adenovirus	1 (1.5%)
Coronavirus HKU1	3 (4.6%)
Coronavirus NL63	2 (3.1%)
Coronavirus OC43	2 (3.1%)
Human Metapneumovirs	8 (12.3%)
Rhinovirus/Enterovirus	21 (32.3%)
Influenza A	6 (9.2%)
Influenza A H1 2009	2 (3.1%)
Influenza A H1	1 (1.5%)
Influenza A H3	3 (4.6%)
Influenza B	7 (10.8%)
Parainfluenza 1	2 (3.1%)
Parainfluenza 4	1 (1.5%)
Respiratory Syncytial Virus	6 (9.2%)
B. pertussis	1 (1.5%)

Supplementary Table 3. Top classification parameters for prediction of disease classes and disease severity for elastic net models, based on train/test/validation (SARS-CoV-2 infection and COVID severity), and train/test (hospitalization duration). Parameters chosen for best-performing median prediction accuracy (AUC for case-control, R2 for duration and severity) across the 100 iterations of training and 10-fold cross-validation. Within best α, λ and number of nonzero probes chosen across the range of best-fitting parameters, including median and IQR values for each. Prediction accuracy (AUC for SARS-COV-2 status, R2 for duration and severity) reported in percentages.

Outcome	Best α	Median (IQR) λ	Median (IQR) Probes	Max Pred	Median (IQR) Pred
SARS-CoV-2	0.4	0.095 (0.037-0.134)	142 (82-272)	97.6	93.4 (92.2-94.7)
Hospital duration	0.1	20.8 (19.0-21.8)	158 (152-174)	15.1	9.62 (8.20-11.2)
Severity	0.01	2.03 (0.52-5.01)	2803 (2346-3032)	53.2	34.8 (26.3-43.2)
