# Supplementary material for: Host methylation predicts SARS-CoV-2 infection and clinical outcome
Source: Commun Med (Lond). 2021 Oct 26;1:42. doi: 10.1038/s43856-021-00042-y (PMC8767772; doi:10.1038/s43856-021-00042-y)
Supplement: Supplementary file 8 — Description of Additional Supplementary Files [file 43856_2021_42_MOESM8_ESM.pdf]

Description of Additional Supplementary Files

File Name: Supplementary Data 1

Description: Custom Illumina Infinium methylation probes included on arrays. Includes probe ID, chromosomal mapping, overlapping genes, transcripts, and transcript features from NCBI, probe type information, if the probe maps to multiple chromosomal loci, and whether the probe was included in manuscript analysis.

File Name: Supplementary Data 2

Description: 13,033 significant CpGs from SARS-CoV-2+ vs SARS-CoV-2- EWAS. Includes probe ID, chromosomal mapping, overlapping genes, and p-value, FDR-adjusted ¬p¬-value (q-value), coefficient, test statistic, and standard error from modeling, as well as average beta value for each sample group.

File Name: Supplementary Data 3

Description: 1,501 significant CpGs from SARS-CoV-2+ vs other upper respiratory infection EWAS. Includes probe ID, chromosomal mapping, overlapping genes, and p-value, FDR-adjusted ¬p¬-value (q-value), coefficient, test statistic, and standard error from modeling, as well as average beta value for each sample group.

File Name: Supplementary Data 4

Description: 516 significant CpGs from other upper respiratory infection vs SARS-CoV-2- EWAS. Includes probe ID, chromosomal mapping, overlapping genes, and p-value, FDR-adjusted ¬p¬-value (q-value), coefficient, test statistic, and standard error from modeling, as well as average beta value for each sample group.

File Name: Supplementary Data 5

Description: Source Data for Figure 2.

File Name: Supplementary Data 6

Description: Source Data for Figure 4.
