# Supplementary material for: Host methylation predicts SARS-CoV-2 infection and clinical outcome
Source: Commun Med (Lond). 2021 Oct 26;1:42. doi: 10.1038/s43856-021-00042-y (PMC8767772; doi:10.1038/s43856-021-00042-y)
Supplement: Supplementary file 9 — Reporting Summary [file 43856_2021_42_MOESM9_ESM.pdf]

nature research

Corresponding author(s): Kathleen C. Barnes

Last updated by author(s): 9/21/2021

Reporting Summary

Nature Research wishes to improve the reproducibility of the work that we publish. This form provides structure for consistency and transparency in reporting. For further information on Nature Research policies, see our Editorial Policies and the Editorial Policy Checklist.

Statistics

For all statistical analyses, confirm that the following items are present in the figure legend, table legend, main text, or Methods section.

n/a	Confirmed	
☐	☒	The exact sample size (n) for each experimental group/condition, given as a discrete number and unit of measurement
☐	☒	A statement on whether measurements were taken from distinct samples or whether the same sample was measured repeatedly
☐	☒	The statistical test(s) used AND whether they are one- or two-sided<br>*Only common tests should be described solely by name; describe more complex techniques in the Methods section.*
☐	☒	A description of all covariates tested
☐	☒	A description of any assumptions or corrections, such as tests of normality and adjustment for multiple comparisons
☐	☒	A full description of the statistical parameters including central tendency (e.g. means) or other basic estimates (e.g. regression coefficient) AND variation (e.g. standard deviation) or associated estimates of uncertainty (e.g. confidence intervals)
☐	☒	For null hypothesis testing, the test statistic (e.g. F, t, r) with confidence intervals, effect sizes, degrees of freedom and P value noted<br>*Give P values as exact values whenever suitable.*
☒	☐	For Bayesian analysis, information on the choice of priors and Markov chain Monte Carlo settings
☒	☐	For hierarchical and complex designs, identification of the appropriate level for tests and full reporting of outcomes
☐	☒	Estimates of effect sizes (e.g. Cohen's d, Pearson's r), indicating how they were calculated

Our web collection on statistics for biologists contains articles on many of the points above.

Software and code

Policy information about availability of computer code

Data collection: *Provide a description of all commercial, open source and custom code used to collect the data in this study, specifying the version used OR state that no software was used.*

Data analysis: Raw array data was processed using seSAMe 1.7.6 in R 4.0.1. EWAS was carried out using GLINT 1.0.4 on the command line. Machine learning analyses were done using Glmnet v2.0-18 and Data.table v1.11.4 in R 3.5.1. Plotting and consolidation was done in R 4.1.0 using ggplot2 v2_3.3.3 and Data.table v1.14.0. All packages are available through CRAN and Bioconductor.

For manuscripts utilizing custom algorithms or software that are central to the research but not yet described in published literature, software must be made available to editors and reviewers. We strongly encourage code deposition in a community repository (e.g. GitHub). See the Nature Research guidelines for submitting code & software for further information.

Data

Policy information about availability of data

All manuscripts must include a data availability statement. This statement should provide the following information, where applicable:

- Accession codes, unique identifiers, or web links for publicly available datasets
- A list of figures that have associated raw data
- A description of any restrictions on data availability

The datasets generated during the current study are available in the Gene Expression Omnibus repository (accession GSE167202) and include original .idat array files and the final processed data matrix for DNA methylation analyses. Source data used to generate Figures 2 and 4 are available as Supplementary Data 5-6.

Field-specific reporting

Please select the one below that is the best fit for your research. If you are not sure, read the appropriate sections before making your selection.

☒ Life sciences ☐ Behavioural & social sciences ☐ Ecological, evolutionary & environmental sciences

For a reference copy of the document with all sections, see nature.com/documents/nr-reporting-summary-flat.pdf

Life sciences study design

All studies must disclose on these points even when the disclosure is negative.

Sample size	Sample size was not pre-determined. All eligible participants were included in the study.
Data exclusions	Samples were excluded due to failed methylation array QC (n = 108), duplication (n = 9), and no SARS-CoV-2 test results (n = 2). Methylation probes with detection p-values > 0.05 were removed, as well as probes overlapping SNPs with global MAF >1% in dbSNP, probes with poor mapping, probes containing non-unique sequence, and probes mapping to non-CpG sites. Probes with >25% missingness were removed.
Replication	Replication was not performed.
Randomization	Plates and methylation BeadChips were balanced for diagnosis and sex. Furthermore, relevant covariation is included in the EWAS model.
Blinding	Blinding was not performed nor relevant to this study.

Reporting for specific materials, systems and methods

We require information from authors about some types of materials, experimental systems and methods used in many studies. Here, indicate whether each material, system or method listed is relevant to your study. If you are not sure if a list item applies to your research, read the appropriate section before selecting a response.

Materials & experimental systems

n/a	Involved in the study	
☒	☐	Antibodies
☒	☐	Eukaryotic cell lines
☒	☐	Palaeontology and archaeology
☒	☐	Animals and other organisms
☐	☒	Human research participants
☒	☐	Clinical data
☒	☐	Dual use research of concern

Methods

n/a	Involved in the study	
☒	☐	ChIP-seq
☒	☐	Flow cytometry
☒	☐	MRI-based neuroimaging

Human research participants

Policy information about studies involving human research participants

Population characteristics	Patients had a mean age 54.2±18.3, were 47.8% female, 51% White, 17.5% Black or African American, 3.4% Asian, and 23.8% identified as other.
Recruitment	Subjects were consented to University of Colorado Emergency Medicine Specimen Bank at the UCHealth Emergency Department and leftover samples from patients tested for COVID-19 were included in this analysis.
Ethics oversight	Colorado Multiple Institutional Review Board (COMIRB #17-1642 The Emergency Medicine Specimen Bank, and COMIRB#20-2021, The 'Omics of COVID-19 Clinical Outcomes)

Note that full information on the approval of the study protocol must also be provided in the manuscript.
